# Purification and Characterization of Chitinases from Ridgetail White Prawn *Exopalaemon carinicauda*

**DOI:** 10.3390/molecules20021955

**Published:** 2015-01-26

**Authors:** Jing Wang, Jiquan Zhang, Fengge Song, Tianshu Gui, Jianhai Xiang

**Affiliations:** 1 Key Laboratory of Experimental Marine Biology, Institute of Oceanology, Chinese Academy of Sciences, Qingdao 266071, China; E-Mails: wangjing926453@gmail.com (J.W.); xiangyangge2@163.com (F.S.); guitianshu@hust.edu.cn (T.G.); jhxiang@qdio.ac.cn (J.X.); 2 University of Chinese Academy of Sciences, Beijing 100039, China

**Keywords:** chitinase, enzymatic characterization, *Exopalaemon carinicauda*

## Abstract

In this paper, we purified two native chitinases from the hepatopancreas of the ridgetail white prawn *Exopalaemon carinicauda* by using ion-exchange resin chromatography (IEC) and gel filtration. These two chitinases, named EcChi1 and EcChi2, were identified by chitinolytic activity assay and LC-ESI-MS/MS. Their apparent molecular weights were 44 kDa and 65 kDa as determined by sodium dodecyl-sulfate polyacrylamide gel electrophoresis (SDS-PAGE). The specific activity of EcChi1 and EcChi2 was 1305.97 U·mg^−1^ and 28.69 U·mg^−1^. The optimal temperature and pH of EcChi1 were 37 °C and pH 4.0, respectively. Co^2+^, Fe^3+^, Zn^2+^, Cd^2+^, and Cu^2+^ had an obvious promoting effect upon chitinase activity of EcChi1. For colloidal chitin, the K_m_ and V_max_ values of EcChi1 were 2.09 mg·mL^−1^ and 31.15 U·mL^−1^·h^−1^.

## 1. Introduction

Chitin is the second most abundant organic compound on Earth. It is widely spread in various organisms and almost 10% of the global landings of aquatic products consist of organisms rich in chitinous materials [1]. More than 80,000 metric tons of chitin is obtained per year from the marine waste. Chitin has been used as an important biomaterial in many areas, such as agriculture, food technology, material science, wastewater treatment, bionanotechnology, and biomedicine [2] since it was discovered. Furthermore, chitooligosaccharides, the degradation products of chitin, have high application value in many fields such as food processing, medicines, and cosmetics [3,4,5]. Among them, the most remarkable application of chitooligosaccharides is human healthcare. Numerous papers have reported that chitooligosaccharides can inhibit tumor growth, treat asthma, increase bone strength, prevent malaria and be used as vectors for delivery of genes in gene therapy [4,6,7].

Chitinases (EC3.2.1.14), a member of the *O*-glycoside hydrolase superfamily, have shown immense potential for hydrolyzing chitin in the most economical way. Specific combinations of chitinolytic enzymes are necessary to obtain the desired chain length of the resulting oligomer [8]. For example, the production of chitooligosaccharides requires high levels of endochitinase activity and low levels of *N*-acetyl-glucosaminidase and exochitinase activity, whereas the production of GlcNAc requires a higher proportion of exochitinase and *N*-acetyl-glucosaminidase activity [8,9]. In addition, chitinases also have other applications including production of single-cell protein, isolation of protoplasts, pest control and genetic engineering for plant fungal disease resistance [1,8].

Abundant research related to chitinases has been done in microorganisms, plants and insects [10,11,12,13,14,15,16]. Studies of chitinases in crustaceans, by contrast, are much fewer. The exoskeleton of crustaceans is rich in chitin, which can comprise 20%–58% of the dry weight of the outer shell [17]. During the molting cycle of crustaceans, chitinases play a significant role by degrading the old chitin into chitooligosaccharides and synthesizing new chitin [18,19,20,21,22,23]. At present, some chitinases in crustaceans, including *Uca pugilator* [23], *Marsupenaeus japonicas* [24,25,26], *Penaeus monodon* [27,28,29], *Fenneropenaeus chinensis* [30], *Litopenaeus vannamei* [31] and *Pandalopsis japonica* [32] have been reported. However, most of these studies were focus on cloning and expression analysis of chitinase genes other than isolation and characterization of native enzymes. In this paper, we extracted two native chitinases (EcChi1 and EcChi2) from the hepatopancreas of *E. carinicauda*. The enzymatic characteristics and kinetic parameters of the chitinase with higher activity (EcChi1) were studied.

## 2. Results and Discussions

### 2.1. Protein Purification, Molecular Weight Determination and Amino Acid Sequence

**Figure 1:**
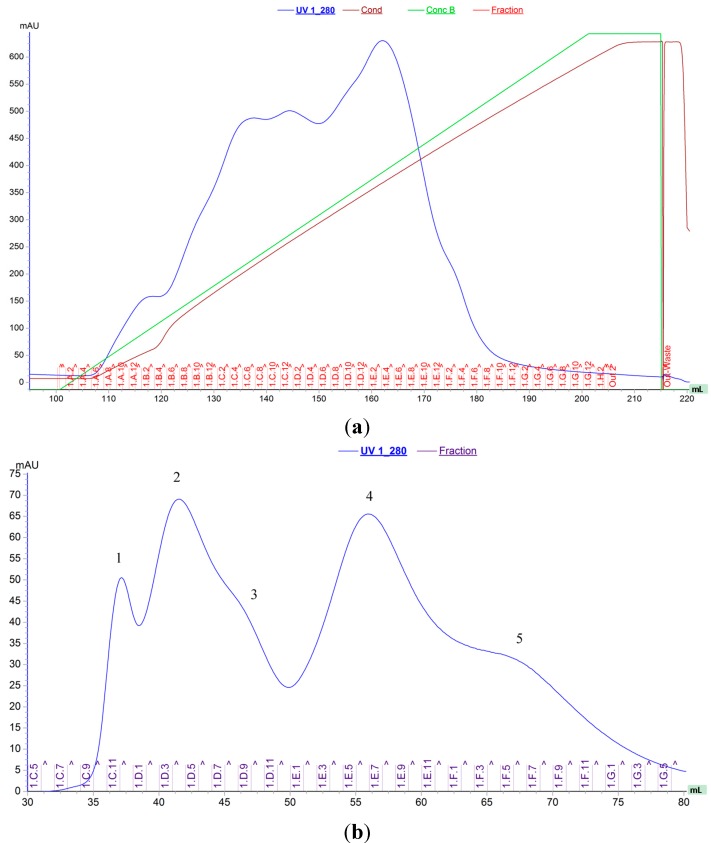
Anion-exchange and gel filtration chromatograms of the *E. carinicauda* chitinases. (**a**) Anion-exchange chromatography on HiTrap Q FF 5 mL. Fractions (1C2 to 1D6) with chitinolytic activity were collected for next process; (**b**) Gel filtration on HiPrep16/60 Sephacryl S-100. Five proteins were purified, chitinolytic activity was detected in peak 4 and 3, and they were named as EcChi1 and EcChi2, respectively.

**Figure 2:**
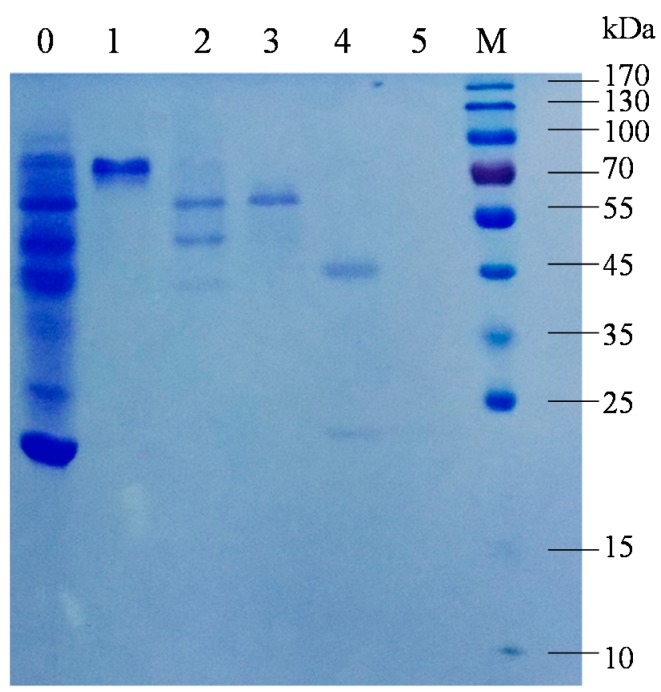
SDS-PAGE analysis of purified proteins. Lane 0, proteins before gel filtration; Lane 1–5, proteins collected after gel filtration, the order number was in accordance with gel filtration chromatogram (Lane 3, EcChi2; Lane 4, EcChi1); Lane M, Molecular weight makers.

**Figure 3:**
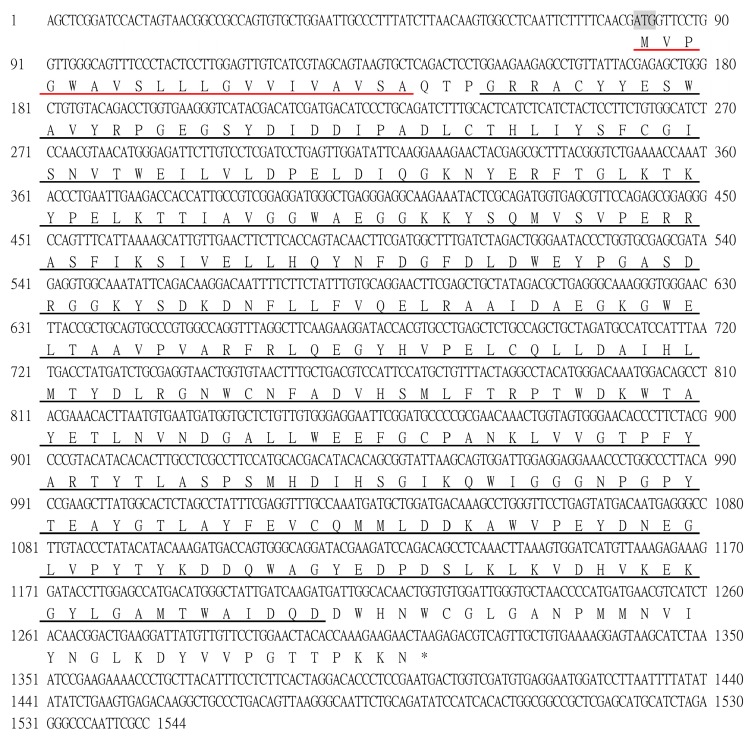

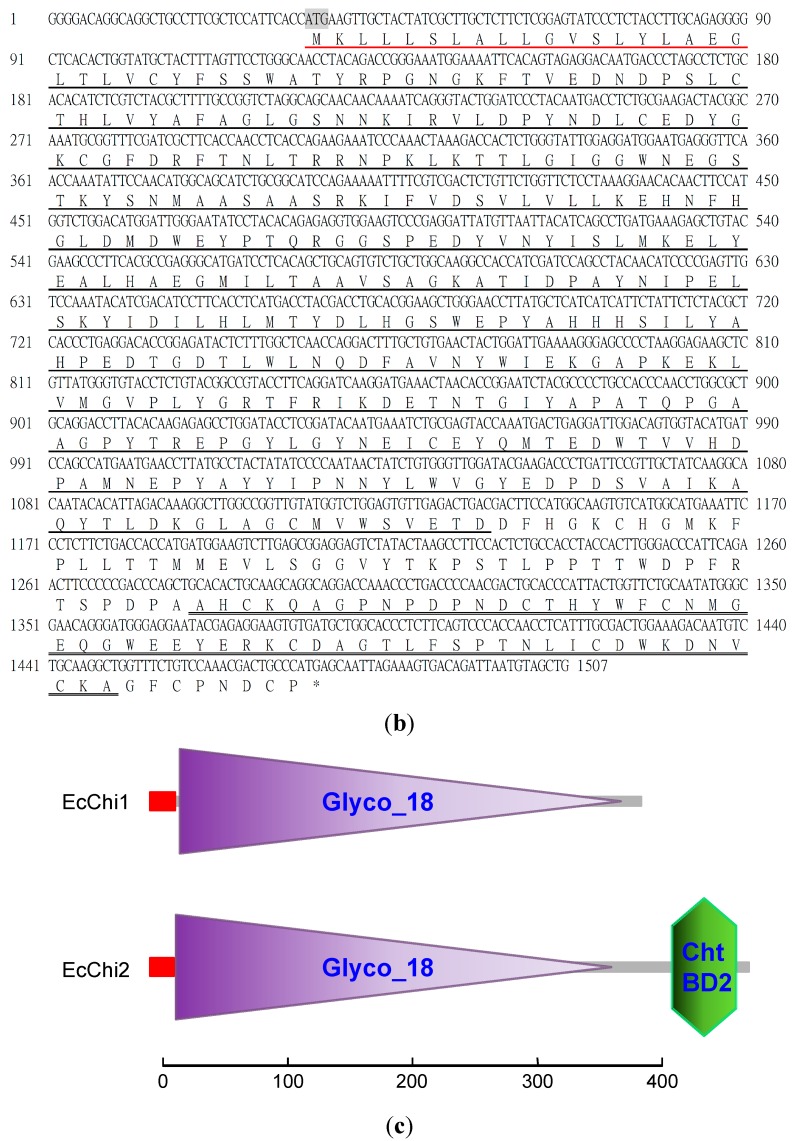
Nucleotide and deduced amino acid sequences of EcChi1 and EcChi2 genes. (**a**) EcChi1; the letters in shadow indicate initiation codon (ATG) and asterisk indicate termination codon (TAA). The signal peptide is underlined with a red single line, and the Glyco_18 domain is underlined with a black single line; (**b**) EcChi2; the letters in shadow indicate initiation codon (ATG) and asterisk indicate termination codon (TGA). The signal peptide is underlined with a red single line, and the Glyco_18 domain is underlined with a black single line, Cht BD2 is marked with a double underline; (**c**) Schematic representation of EcChi1 and EcChi2 protein structures.

Two proteins with chitinolytic activity, named EcChi1 and EcChi2, were purified from hepatopancreas of *E. carinicauda* by anion-exchange chromatography and gel-filtration chromatography (Figure 1). According to the chromatogram, the amount of EcChi1 was much higher than that of EcChi2, which was consistent with chitinolytic activity detection. Determined by SDS-PAGE, the apparent molecular weight of EcChi1 and EcChi2 was estimated to be 44 kDa and 65 kDa (Figure 2). Based on the LC-ESI-MS/MS results and *E. carinicauda* transcriptome (ECT) database, the purified EcChi1 and EcChi2 with chitinolytic activity were definitely chitinases. Based on ECT data and SMART RACE method, we obtained the full-length cDNA sequences of EcChi1 and EcChi2. The full-length nucleotide sequences and the deduced amino acid sequences are shown in Figure 3a,b. The ORF of EcChi1 and EcChi2 encoded 410 and 480 amino acids with a predicted molecular weight of about 46,346.44 Da and 53,918.87 Da, respectively. The theoretical isoelectric points (PI) of EcChi1 and EcChi2 were 4.83 and 5.00. Both of the deduced amino acid sequences of EcChi1 and EcChi2 contain signal peptide and glycosyl hydrolases family 18 (Glyco_18) domains. In addition, there is also one type 2 chitin-binding domain in the amino acid sequence of EcChi2 following Glyco_18 domain (Figure 3c). No protein with chitinolytic activity was purified from hepatopancreas of *E. carinicauda* by cation exchange resin (HiTrap CM FF).

### 2.2. Chitinolytic Activity, Protein Concentration and the Specific Activity

The chitinolytic activity of EcChi1 and EcChi2 was 21.80 U·mL^−1^ and 8.28 U·mL^−1^. The protein concentration of EcChi1 and EcChi2 was 0.0167 mg·mL^−1^ and 0.289 mg·mL^−1^, respectively. The specific activity of EcChi1 and EcChi2 was 1305.97 U·mg^−1^ and 28.69 U·mg^−1^, respectively.

### 2.3. Characterization of Purified EcChi1

**Figure 4:**
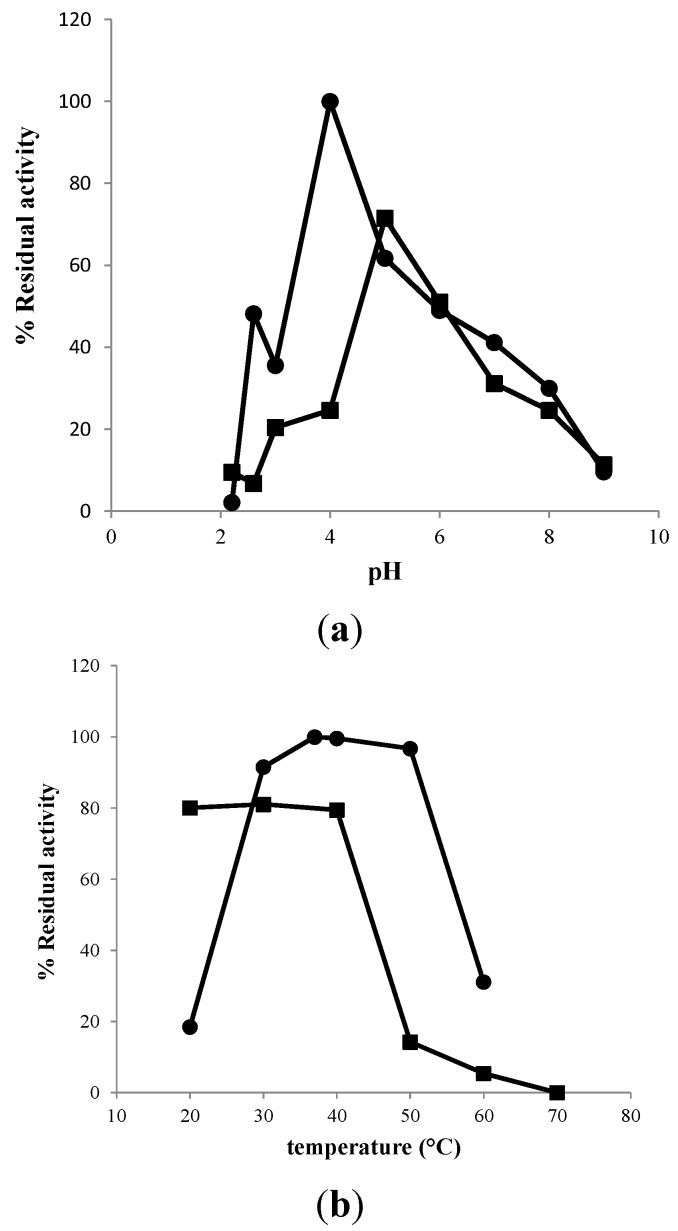
(**a**) Effects of pH on chitinolytic activity (●) and stability (■) of EcChi1; (**b**) Effects of temperature on chitinolytic activity (●) and stability (■) of EcChi1.

The results above showed that the purity and amount of EcChi1 was much higher than that of EcChi2. Hence, the purified EcChi1 was selected to study its enzymatic characteristics, including the optimal pH and temperature, effects of metal ions and enzyme stabilities. The results showed that EcChi1 had maximal chitinolytic activity at pH 4.0 (Figure 4a) and was most stable at pH 5.0 with the residual activity was above 70% after incubated for 24 h (Figure 4a). The *P. japonicas* chitinase purified from the liver [33] and *L. vannamei* chitinase purified from the digestive gland [34] had an optimal activity at pH 6.7 and pH 6.5, respectively, and were adapted to an acidic environment like EcChi1.

To determine the optimal temperature and thermal stability of EcChi1, the chitinolytic activity at different temperatures (from 20 to 60 °C) was measured. The results indicated that the apparent optimal temperature of EcChi1 was 37 °C (Figure 4b) which was 10–15 °C lower than that of most other shrimp chitinases reported so far [21,33], but almost as low as that of *Meganyctiphanes norvegica* [35]. The enzyme was stable below 40 °C, and retained above 85% activity after incubation for 24 h. However, its activity had an obviously decreased when the enzyme was incubated in 50 °C or 60 °C for 24 h (Figure 4b). This is quite different from the chitinases of *E. superba* and *M. norvegica*, which were 50% inactivated after 60 min incubation at 46 °C [35].

**Figure 5:**
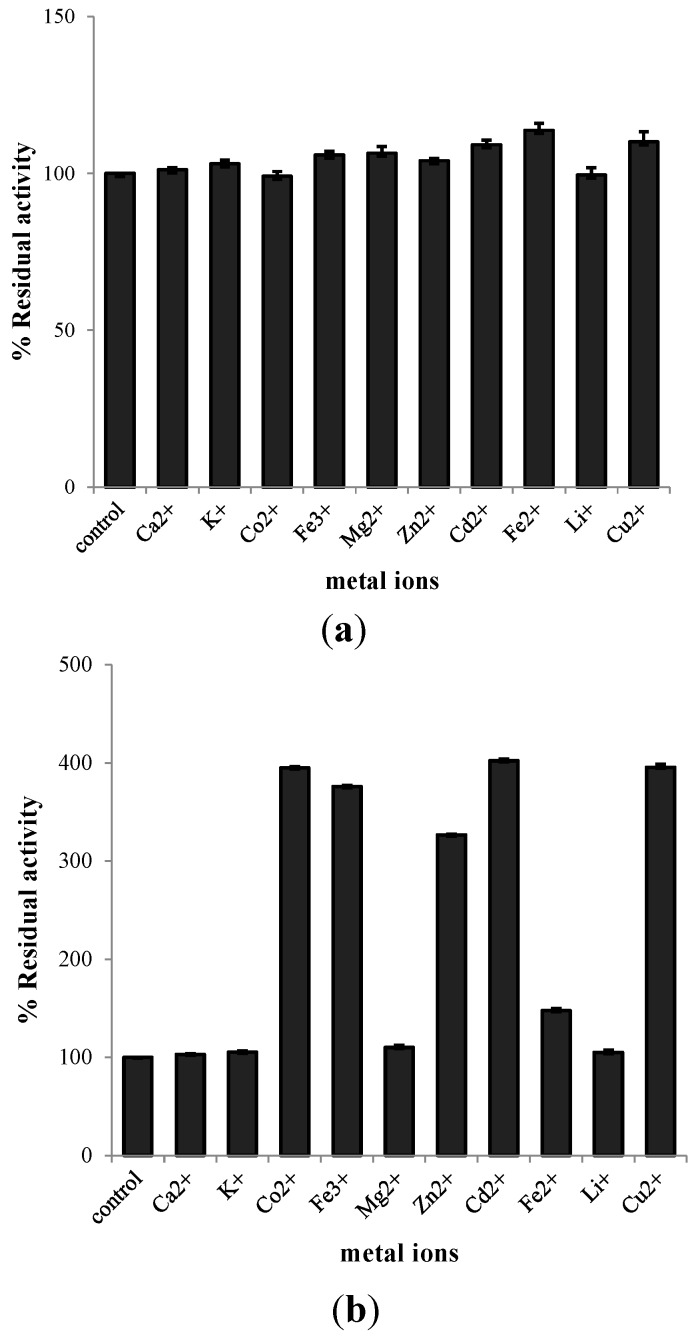
(**a**) Effect of 10 mM metal ions on chitinolytic activity of EcChi1; (**b**) Effect of 10 mM metal ions on chitinolytic activity of EcChi1 after incubated with EcChi1 for 20 h.

Ten kinds of metal ions were used to analyze their effect on chitinolytic activity of EcChi1. No metal ions showed significant effects on enzyme activity when immediately added to the reaction mixture (Figure 5a). However, some metal ions, Co^2+^, Fe^3+^, Zn^2+^, Cd^2+^ and Cu^2+^ had an obvious promoting effect upon chitinase activity of EcChi1 when incubated with enzyme for 20 h. Compared to the control, the chitinase activity of EcChi1 with these ions were increased 300%–400%. Besides, Fe^2+^ also had a certain positive effect on EcChi1 activity. The effect of other ions on EcChi1 activity was similar to that of the control (Figure 5b). This result was different from that of other crustaceans chitinases; for instance, the chitinase activity of P. japonicas was inhibited by Zn^2+^ and the residual activity was below 46% [33].

### 2.4. Kinetic Parameters for Colloidal Chitin Hydrolysis by EcChi1

**Figure 6:**
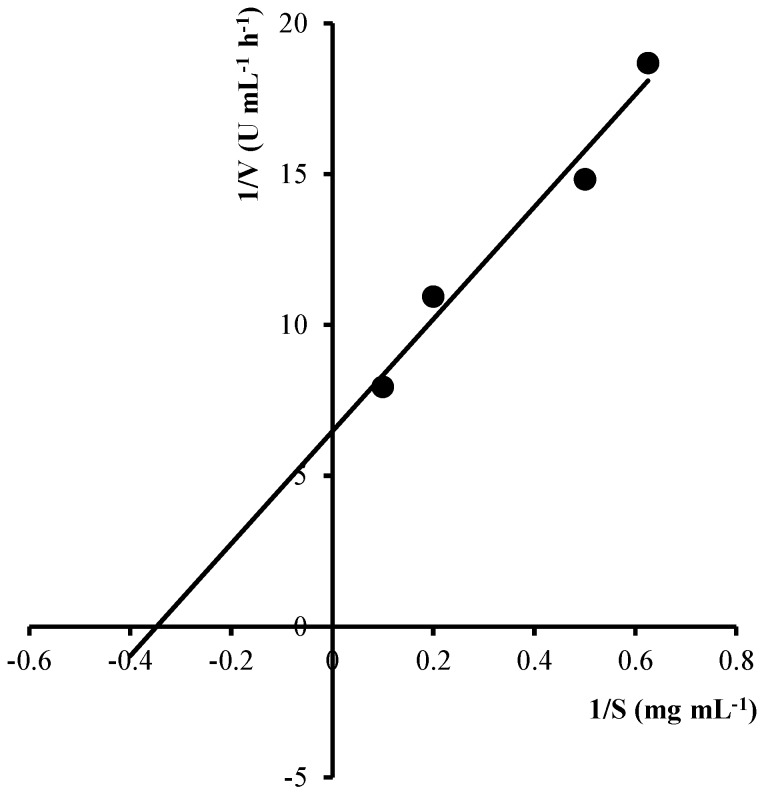
Effect of colloidal chitin concentration on EcChi1 activity. The data were expressed in a Lineweaver-Burk plot.

For colloidal chitin, the K_m_ and V_max_ values of the purified EcChi1 was 2.09 mg·mL^−1^ and 31.15 U·mL^−1^·h^−1^, respectively, which was calculated from the Lineweaver-Burk’s plot (Figure 6).

## 3. Experimental Section

### 3.1. Materials

The ridgetail white prawns, *Exopalaemon carinicauda* with a body length of 5.5 ± 0.5 cm were bred in our laboratory. HiTrap ion exchange resins (HiTrap Q FF, 1 mL; HiTrap CM FF, 1 mL), Gel filtration resins (HiPrep 16/60 Sephacryl S-100 High Resolution), HiPrep Desalting resins (HiPrep 26/10 Desalting Sephadex G-25 Fine) were purchased from GE Healthcare (Uppsala, Sweden). Chitin (from shrimp shells) was purchased from Sigma (St. Louis, MO, USA), and colloidal chitin was prepared using the reported method [36]. 4-(Dimethylamino)-benzaldehyde (DMAB) and potassium tetraborate tetrahydrate were purchased from Sangon Biotech (Shanghai, China). *N*-Acetyl-D-glucosamine was purchased from Life Science (St. Louis, MO, USA).

### 3.2. Chitinolytic Activity Assay

The chitinolytic activity was measured by quantitative estimation of the reducing sugars released during the hydrolysis of colloid chitin via the method of Reissig* et al.* [37]. *N*-Acetyl-d-glucosamine was used as the standard. The reaction mixture included 50 μL 1% colloidal chitin, 50 μL acetate buffer (pH 5.0, 50 mM) and 50 μL enzyme solution. After digestion for 2 h at 37 °C, the reaction was terminated by centrifuged at 4000 rpm, for 10 min. 25 μL 1% (w/v) potassium tetraborate was added to 50 μL supernatant of the standard or samples, respectively. The tubes were heated in the boiling water bath for 7 min and cooled rapidly in cold water. Then 250 μL acetic acid and 125 μL DMAB were added to each tube and all tubes were placed in a water bath at 37 °C after mixing. Twenty minutes later, all tubes were cooled in cold water and the absorbance measured at 585 nm. Blank (control assay) was done with inactivated enzymes. One unit of chitinase activity was defined as the amount of enzyme, which releases 1 μg of *N*-acetyl-d-glucosamine per hour from colloid chitin under the conditions described above.

### 3.3. Protein Concentration Determination

The protein concentrations were determined by the method of Bradford [38]. Various amounts of 100 μg·mL^−1^ BSA (2–12 μg) were used to draw standard calibration curve. The specific activity was expressed as units of activity per milligram of protein (U·mg^−1^ of protein).

### 3.4. Isolation and Purification of Chitinases from E. carinicauda (EcChis)

The hepatopancreas were dissected from 100 ridgetail white prawns and incubated by Tris solution (pH 11.5, 50 mM) for 1 h at 4 °C. The mixture was centrifuged at 10,000 rpm, 4 °C for 15 min and the precipitate was discarded. The pH of the supernatant which contained crude protein was adjusted to pH 7.0 with 1 M HCl.

The isolation and purification of chitinases was performed in three steps. The supernatant was precipitated with ammonium sulfate at 70% saturation, and kept at 4 °C overnight. Then the precipitated proteins were collected by centrifugation at 10,000 rpm, 4 °C and resuspended by 30 mL Na_2_HPO_4_-NaH_2_PO_4_ buffer (pH 7.2, 20 mM). And it was desalted with HiPrep Desalting resins. During purification process, chitinase activity was assayed using the method described above.

The total protein containing chitinase was isolated by using anion exchange resin (HiTrap Q FF) and cation exchange resin (HiTrap CM FF) in sequence. The resins were equilibrated with 20 mM, pH7.2 Na_2_HPO_4_-NaH_2_PO_4_ buffer, and the adsorbed protein was eluted by linear gradient with sodium phosphate buffer containing 1 M NaCl. The fractions with high elution peak were collected and its chitinolytic activity measured. The high chitinase activity fractions was gathered, desalted and concentrated.

Then, the concentrated sample was subjected to a gel filtration resin (HiPrep 16/60 Sephacryl S-100 High Resolution). During the process, 50 mM, pH 7.2 sodium phosphate buffer contained 0.15 M NaCl was adopted, the amount of sample application was 2 mL, and the procedure was run at a flow rate of 1 mL·min^−1^. One milliliter per fraction was collected. The chitinase activity of each elution peak was determined.

### 3.5. Identification of Purified EcChis by LC-ESI-MS/MS

The molecular mass of each purified protein was estimated by sodium dodecyl sulfate polyacrylamide gel electrophoresis (SDS-PAGE) using 12% separating gel and 5% stacking gel. The gel was stained with Coomassie brilliant blue.

The bands with chitinolytic activity were excised from gel for protein identification via liquid chromatography electrospray ionization tandem mass spectrometry (LC-ESI-MS/MS). The Mascot software was used to analyze the mass spectrometric data combined with *E. carinicauda* transcriptome (ECT) database [39].

### 3.6. Effect of pH Value on Chitinolytic Activity

The optimal pH of the purified chitinase was determined by varying the pH of reaction mixture from 2.2 to 9.0. The buffer used to generate pH ranges were 50 mM glycine-HCl buffer (pH 2.2–3.0), sodium acetate buffer (pH 4.0–5.0), sodium phosphate buffer (pH 6.0–8.0), and sodium carbonate buffer (pH 9.0).

The stability of chitinase activity at different pH was also determined. The purified chitinase was incubated with different pH buffers mentioned above at 37 °C for 24 h without substrate, respectively. After incubation, the residual activity of the treated enzyme was measured. The activity at the optimal pH without incubation was taken as 100%.

### 3.7. Effect of Temperature on Chitinolytic Activity

The measurement of the optimum temperature was conducted by putting reaction mixtures in different temperatures (ranging from 20 to 60 °C) at the optimal pH condition and the amount of reducing sugars released from colloid chitin was measured.

To determine the thermal stability, chitinase was incubated at different temperature (ranging from 20 to 70 °C) for 24 h. Then the residual activity was measured as described in Section 3.2. The activity at the optimal temperature without incubation was taken as 100%.

### 3.8. Effect of Metal Ions on Chitinolytic Activity

Several metal ions (Ca^2+^, Mg^2+^, Cu^2+^, Li^+^, Fe^2+^, Fe^3+^, K^+^, Cd^2+^, Co^2+^ and Zn^2+^) were used to identify their effect on chitinolytic activity. Each metal ion was added into reaction mixture with a final concentration at 10 mM, and then the chitinolytic activity was determined immediately following the method described above.

To test the enzyme stability to metal ions, the chitinase was incubated with metal ions (total reaction volume of 70 μL) for 20 h at 4 °C. A control assay was done in the reaction without metal ions and its activity was taken as 100%.

### 3.9. Kinetic Parameters for Colloidal Chitin Hydrolysis by EcChi1

To determine the kinetic constants (K_m_ and V_max_) of EcChi1, different concentrations of substrate (colloidal chitin) ranging from 0.2 to 10 mg·mL^−1^ was mixed with 50 μL enzyme in sodium acetate buffer (pH 4.0, 50 mM), and incubated at 37 °C for 2 h. The activity was measured as described in Section 3.2. Then, the K_m_ and V_max_ values were determined by Lineweaver-Burk plot.

## 4. Conclusions

In this study, two chitinases, EcChi1 and EcChi2, were isolated from hepatopancreas of *E. carinicauda*. SDS-PAGE gel showed that the apparent molecular weight were 44 kDa and 65 kDa, respectively. Some biochemical and enzymatic properties of EcChi1 were characterized. The optimal pH, optimal temperature, pH stability, and thermal stability of EcChi1 were pH 4.0, 37 °C, pH 5.0, and below 40 °C. The activities of EcChi1 were strongly activated by Co^2+^, Fe^3+^, Zn^2+^, Cd^2+^, and Cu^2+^. And EcChi1 exhibited K_m_ values of 2.09 mg·mL^−1^ and V_max_ values of 31.15 U·mL^−1^·h^−1^ when using colloidal chitin as substrate. These results will provide a certain basis for the application of EcChis in the future.
